# Use of Mechanical Enhanced Colonoscopy to Improve Polyp Detection During Colorectal Cancer Screening: A Real-World Healthcare Database Analysis

**DOI:** 10.3390/jcm14176346

**Published:** 2025-09-08

**Authors:** Abraham Z. Cheloff, Seth A. Gross

**Affiliations:** NYU Langone Health, 550 1st Avenue, New York, NY 10016, USA; abraham.cheloff@nyulangone.org

**Keywords:** Endocuff, colonoscopy, colonic polyp, colonic adenoma

## Abstract

**Introduction:** High performance colonoscopy requires the monitoring of an individual’s adenoma detection rate (ADR). The Endocuff (EndoCuff Vision, Olympus America Inc., Center Valley, PA, USA) is an endoscopic distal attachment device that increases surface area exposure during colonoscopy. While studies have shown that Endocuff increased ADR, real-world data is limited on its effectiveness. **Methods:** The Premiere Health Database was reviewed from 2018 to 2021 to identify patients 50 years of age or older who had a screening colonoscopy. A keyword search for “Endocuff” was used to determine if Endocuff was utilized, and ICD10 codes were analyzed to determine if a polyp was found. Our primary outcome was a polyp detection rate (PDR) for Endocuff-assisted colonoscopy (EAC) and standard colonoscopy (SC). Secondary outcomes included an estimated adenoma detection rate (eADR). Logistic regression modeling was performed to examine the difference in PDR between the EAC and SC groups after controlling for baseline characteristics, insurance type, and provider experience. **Results:** Gastroenterologists performed 893,560 screening colonoscopies, of which 0.7% were Endocuff-assisted, while surgeons performed 234,962 screening colonoscopies and 0.5% were Endocuff-assisted. PDR was higher with EAC for both gastroenterologists (72.0% vs. 57.4%) and surgeons (55.6% vs. 43.7%), with eADR following similar trends. The odds ratio of polyp detection with vs. without Endocuff was 1.91 for gastroenterologists and 1.62 for surgeons. After adjusting for patient and provider factors, the adjusted odds ratios are 2.01 and 1.61, respectively. **Conclusions:** While Endocuff utilization remains low, this large study using real-world data demonstrates the ability to improve eADR by over 10% compared to standard colonoscopy.

## 1. Introduction

Colorectal cancer (CRC) is the second leading cause of cancer-related death, with an expected 152,810 new cases in 2024 leading to 53,010 fatalities [1]. This is despite steep declines in the incidence and mortality of CRC over the past 50 years [2]. While multiple options exist for CRC screening, including CT colonography, fecal DNA testing, and flexible sigmoidoscopy, colonoscopy remains the hallmark of screening tools [3,4,5], with decreased incidence and mortality of CRC attributed to the use of colonoscopy [6,7,8,9,10].

However, the performance of colonoscopy is both operator- and specialty-dependent, with higher adenoma detection rates (ADRs) and lower complication rates associated with procedures performed by gastroenterologists [11]. National societies such as the American College of Gastroenterology (ACG) and American Society for Gastrointestinal Endoscopy (ASGE) developed quality metric documents to help decrease variability amongst physicians [12]. Established quality indicators include process indicators for the colonoscopy that are pre-procedure (i.e., split dose bowel preparation), intra-procedure (i.e., ADR, cecal intubation rate), and post-procedure (i.e., use of appropriate surveillance intervals and the proportion of adverse events) [12]. Interventions have been designed to improve these indicators such as lower volume bowel preparation [13], education [14], and operator feedback [15].

There are additional measures designed to assess the outcomes from colonoscopy, the most validated of which is ADR [12]. Increased individual ADR has been associated with a decreased risk of CRC incidence and mortality [16]. Strategies to increase ADR include adequate withdrawal time, inspection technique, computer-aided detection (CADe) such as artificial intelligence, endoscopic attachment devices, among others [17,18,19].

**Figure 1:**
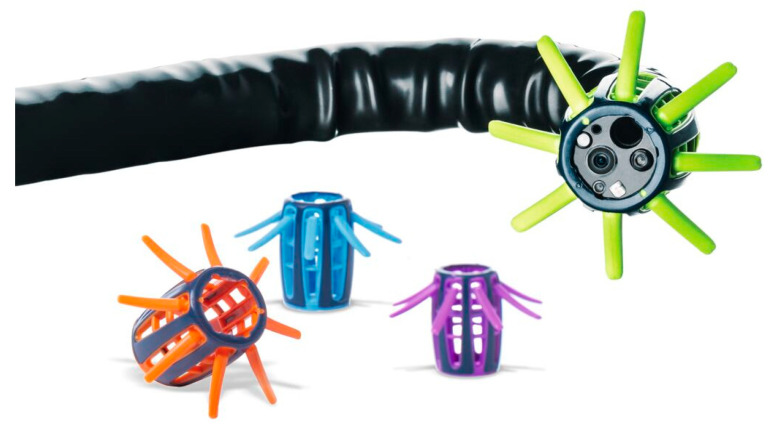
The EndoCuff Vision in small (purple), medium (blue), large (green), and XL (orange). Image provided courtesy of Olympus Corporation of the Americas. All rights reserved.

Endocuff (EndoCuff Vision, Olympus America Inc., Center Valley, PA, USA) is an endoscopic distal attachment device with finger-like projections, increasing surface area exposure of colonic folds [20] (Figure 1). Endocuff has already been shown to increase ADR in randomized clinical trials in the USA, Europe, and Asia [21,22,23,24,25]. Further work has shown that when combined with CADe, ADR with Endocuff is even further increased [26,27], and Endocuff can even improve the ability of artificial intelligence computer-aided detection (CADe) systems to locate polyps, including small or sessile lesions [26,28]. However, there is a lack of real-world data on the use of Endocuff, particularly by surgeons. With promising results from these trials, here we seek to evaluate the effectiveness of Endocuff-assisted colonoscopy (EAC) as compared with standard colonoscopy (SC) in increasing the polyp detection rate (PDR) and estimated ADR using real-world data.

## 2. Methods

### 2.1. Study Design and Inclusion Criteria

We performed a retrospective cohort study of all patients aged 50 and older who underwent colorectal cancer screening via colonoscopy with a gastroenterologist or surgeon. Patients with a family or personal history of colorectal cancer or colorectal polyps were excluded. This study was reported in accordance with the Strengthening the Reporting of Observational Studies in Epidemiology (STROBE) guidelines to ensure comprehensive and transparent reporting.

### 2.2. Data Sources

The Premier Healthcare Database (PHD), a US, service-level hospital-based database containing administrative, financial, and healthcare data for inpatient and outpatient encounters [29], was utilized with claim data from January 2018 to December 2021 (due to data availability). Information in the PHD is de-identified and HIPAA-compliant in accordance with the HIPAA Privacy Rule. International Classification of Disease 10th Revision Diagnostic (ICD10) codes, Current Procedural Terminology (CPT) codes, and Healthcare Common Procedure Coding System (HCPCS) codes were used to identify patients who met the initial inclusion criteria. The initial inclusion criteria were patients who had a primary diagnosis as screening for malignant neoplasm of the colon (ICD-10 Code: Z12.11) and had a colonoscopy procedure (HCPCS codes: G0105, G0121; or CPT codes: 45378, 45380, 45381, 45382, 45384, 45385, 45386, 45388, 45389, 45390, 45398). A keyword search for “Endocuff” from the chargemaster file was used to determine whether EndoCuff was utilized during the procedure. Original Endocuff and Endocuff Vision were not distinguished. Additional ICD10 codes were cross-referenced (D12.X, D1.X, K63.5, K51.4, C18-21.X) to determine if a polyp was found during each colonoscopy and (Z80.0, Z85.038, Z85.048, and Z86.010) if a patient had personal or family history of malignant neoplasm of digestive organs/polyps (Supplementary Table S1). Patient demographic characteristics including age, gender, and race, along with healthcare insurance type and hospital characteristics such as bed size, teaching status, and regions, were collected. Total number of colonoscopies performed in the four-year period was collected for each gastroenterologist and surgeon who performed as least one colonoscopy, as a proxy for the endoscopist experience level. This study was exempt from Institutional Review Board oversight due to the use of de-identified data.

### 2.3. Outcomes

The primary outcome was the polyp detection rate (PDR), defined as the proportion of colonoscopies in which at least one polyp was identified, between the EAC and SC groups. Secondary outcomes include the estimated adenoma detection rate (eADR) as compared between the same groups, absolute risk difference between EAC and SC, and the odds ratio of a polyp being detected with EAC vs. SC.

### 2.4. Statistical Analysis

Baseline patient and provider characteristics were summarized using descriptive statistics. Between-group comparisons were performed using chi-square tests for categorical variables and *t*-tests for continuous variables. Utilization of Endocuff was calculated as the number of cases utilizing Endocuff divided by the total number of cases performed per time period. PDR was calculated for each group as the percent of colonoscopies in which at least one polyp was found. As histology data was not available, eADR was calculated as PDR * adenoma to polyp detection rate quotient (APDRQ), using a value for the latter of 0.72 for screening colonoscopies [30]. Absolute risk difference was calculated as EAC eADR − SC eADR. Number needed to treat (NNT) was calculated as 1 divided by the absolute risk difference. Odds ratios were calculated for the odds of a polyp being detected with EAC vs. SC. Logistic regression was performed in order to calculate an odds ration adjusted for patient demographics, provider experience, and hospital characteristics. Two sensitivity analyses were performed. To reflect the real-world scenario and to adjust the unbalance of sample size between the two groups (with/without Endocuff utilization), we randomly selected a sample that had the same numbers of patients who were in the Endocuff group from the without Endocuff group as a sub-cohort for sensitivity analysis. The second sensitivity analysis was performed on a sub-cohort that was selected by using a propensity score matching (1:3) on patient baseline characteristics and hospital characteristics. Logistic regression modeling was performed to examine the difference in PDR between the EAC and SC groups after controlling for patient baseline characteristics, insurance types, provider experience, and hospital characteristics. No missing data was found in this study. In the Premier database for most data elements, less than one percent of patient records have missing information and for key elements, such as demographics and diagnostic information, less than 0.01 percent have missing data. Data analysis was performed using SAS Version 9.4 (SAS Institute Inc., Cary, NC, USA) and supported by Olympus.

### 2.5. Role of Funding Source

The clinical study sponsor, Olympus Corporation of the Americas, assisted in designing and conducting the statistical analysis.

## 3. Results

### 3.1. Colonoscopy Characteristics and EAC Utilization

**Figure 2:**
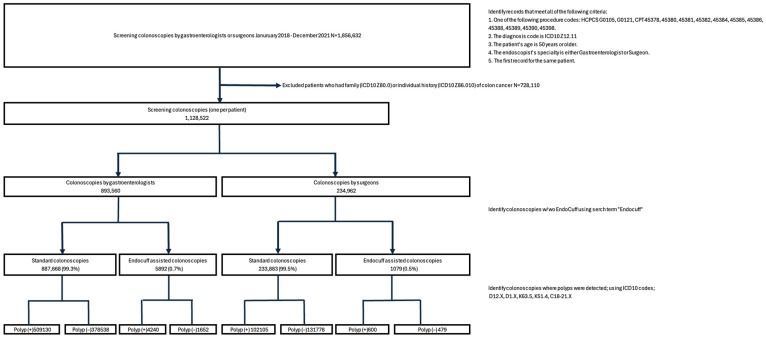
Breakdown of screening colonoscopy type.

**Figure 3:**
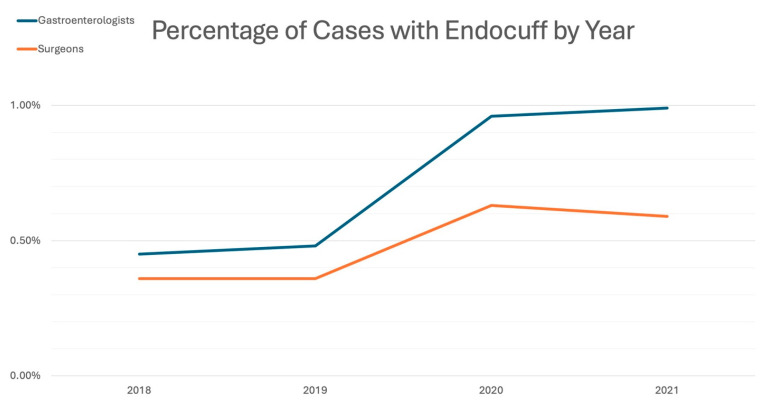
Percentage of cases with Endocuff by year.

Between 2018 and 2021, 1,128,522 screening colonoscopies were identified, of which 893,560 were performed by gastroenterologists and 234,962 were performed by surgeons. Gastroenterologists performed 887,668 (99.3%) standard colonoscopies and 5892 (0.7%) Endocuff-assisted colonoscopies, while surgeons performed 233,883 (99.5%) and 1079 (0.5%), respectively (Figure 2). Endocuff utilization increased yearly, from a low of 0.45% in 2018 to 0.99% in 2021 for gastroenterologists, and from 0.36% to 0.59% for surgeons. The absolute number of EAC rose by year as well, despite the total numbers of colonoscopies falling in 2020 and 2021 (Table 1; Figure 3).

Baseline patient characteristics are presented in Table 2. Age, gender, and race were all statistically different between groups for gastroenterologists, most likely due to the large sample size. However, differences were not clinically meaningful.

### 3.2. Outcomes

**Figure 4:**
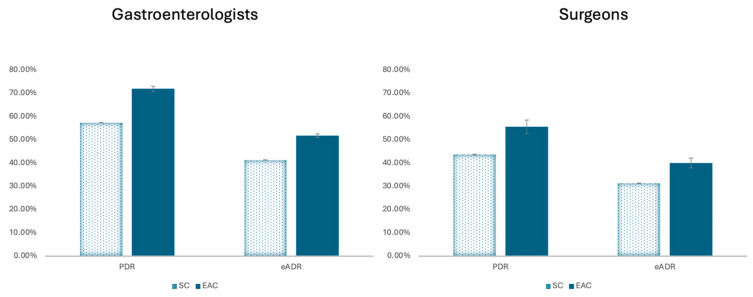
Overall polyp detection rate (PDR) and estimated adenoma detection rate (eADR) for standard colonoscopy (SC) vs. Endocuff-assisted colonoscopy (EAC).

For gastroenterologists, PDR in the SC group was 57.4% (95% CI 57.3–57.5%), while in the EAC group, it was 72.0% (95% CI 70.8–73.1%). eADR was similarly higher in the EAC group (51.8%, 95% CI 51.0–52.6%) than the SC group (41.3%, 95% CI 41.2–41.4%) (Figure 4). The absolute risk difference based on eADR is 10.5%, with a calculated number needed to treat (NNT) of 9.5.

Surgeon’s PDR in the SC group was 43.7% (95%CI 43.5–43.9%) vs. 55.6% (95%CI 52.6–58.6%) in the EAC group. eADR was 31.4% (95%CI 31.3–31.6%) in the SC group and 40.0% (95%CI 37.0–41.2%) in the EAC group. The absolute risk difference based on eADR is 8.5% with an NNT of 11.8.

The unadjusted odds ratio of a polyp being detected with vs. without Endocuff is 1.91 (95%CI 1.80–2.02) for gastroenterologists and 1.62 (95%CI 1.43–1.92) for surgeons. After adjusting for patient demographics, provider experience, and hospital characteristics, the adjusted odds ratio is 2.01 (95%CI 1.90–2.13) for gastroenterologists and 1.61 (95%CI 1.42–1.82) for surgeons.

### 3.3. Sensitivity Analyses

In order to reduce selection bias associated with the unbalanced number of EAC subjects in each group, 6971 patients who underwent standard colonoscopy were randomly selected for subgroup analysis. This group had similar baseline characteristics to the entire standard colonoscopy dataset and remained significantly different from the EAC group. A 1:3 propensity score-matched cohort was also constructed, which did not show statistically significant differences in the baseline age, gender, or race with the EAC group (Table 3).

For all patients, the use of Endocuff was associated with a 96% higher odds of polyp detection (OR 1.96, 95% CI 1.86–2.07, *p* < 0.0001). This significant association remained valid in our random subgroup (OR 1.82, 95% CI 1.68–2.00, *p* < 0.0001) and propensity score-matched subgroup (OR 1.73, 95% CI 1.63–1.83, *p* < 0.0001) though with a smaller effect size. Polyp detection was also strongly associated with the procedure being performed by a gastroenterologist (OR 1.82, 95% CI 1.80–1.84, *p* < 0.0001), which was further validated in our random subgroup (OR 1.90, 95% CI 1.711–2.10, *p* < 0.0001) and propensity score-matched group (OR 1.77, 95% CI 1.65–1.90, *p* < 0.0001).

## 4. Discussion

Despite the fall of colorectal cancer incidence over the past decades with the utilization of screening, there remains significant mortality [1]. While colonoscopy has provided marked improvement in CRC incidence and mortality, the need for continued quality improvement and optimization remains [17].

Our study, using real-world data from over 1,000,000 screening colonoscopies across the United States, found that Endocuff improved PDR and eADR in colonoscopy for colorectal cancer screening performed by both gastroenterologists and surgeons. The eADR of 51.8% when used by gastroenterologists places Endocuff users above the current acceptable standard of 35%, which has continued to rise [12]. The 10.5% absolute risk difference between SC and EAC with an NNT of 9.5 means that, for our populations of 887,668 colonoscopies that were performed using standard technique, we could expect an additional 88,766 colonoscopies to result in at least one adenoma detected if Endocuff was used in all cases. A prior 2020 randomized control trial found an ADR of 53% when using Endocuff [22], further supporting our results. These results are on par with the ADR differences achieved by the Endocuff device in randomized control trials performed in Vietnam [24], with more modest results seen in England [25] and in pooled data including the United States, Italy, Greece, the Netherlands, Germany, and Hong Kong [31]. Population-level data in other communities would be helpful to better compare the utility of Endocuff across continents.

Overall, the utilization of Endocuff remained low with only 0.7% of procedures performed by gastroenterologists and 0.5% of procedures performed by surgeons utilizing Endocuff. This lack of utilization is most likely multifactorial. Data on Endocuff has been mixed, with some studies finding only modest improvement in ADR, especially for those with an already low ADR [32]. The improvement provided by Endocuff is similar to enhanced imaging techniques such as chromoendoscopy and narrow-band imaging that many endoscopists are comfortable using [32], though Endocuff in conjunction with narrow-band imaging can even further increase ADR [33,34]. The advantage of Endocuff can also be operator-dependent, and as with any new device, will require training and comfort by proceduralists. Additionally, given that Endocuff is single-use and incurs a per-procedure cost that may accumulate, widespread adoption would require robust cost-effectiveness data. A decision-analytic Markov model evaluating Endocuff use found cost-savings for both device purchasers and health plans through consistent Endocuff use [35], though further data would help to assuage concerns.

Our study is limited by its retrospective design using a large administrative database which has the potential for coding errors and incomplete documentation. The lack of histopathology results of colonoscopy, and thus eADR being used as a proxy for ADR based on the calculated PDR from claims data, may not reflect true ADR and further limits the generalization of the results to different locations, sizes, and histologies. Unmeasured variables such as bowel preparation or sedation type may also have effects on PDR, and the conversion factor from PDR to eADR has not been specifically validated when using Endocuff. There is also a lack of randomization between those who receive EAC and SC, though EAC has already been shown to be beneficial in randomized trials [21,22]. Endocuff utilization was low (0.7%), decreasing the power of the study and raising the possibility of selection bias. However, our results were affirmed by our sensitivity analysis, which suggested that selection bias was not pervasive in the overall cohort.

Our database is limited to hospital-based colonoscopies and thus limits generalizability to endoscopy centers and private practice, which represents a large proportion of endoscopy practice. Additionally, as the database is based on data from the United States, generalizability may be limited beyond the United States healthcare system. Despite these limitations, our study encompasses data from over 1113 hospitals from a mix of rural and urban areas, as well as non-profit, nongovernmental, community, and teaching hospitals that provide a diverse and widely generalizable population [29].

## 5. Conclusions

This large study investigating Endocuff-assisted colonoscopy demonstrated EAC improved eADR by over 10% compared to standard colonoscopy. Mechanical enhancement plays a valuable role in maximizing surface area exposure of proximal colonic folds to help improve adenoma detection. Overall, the low utilization of Endocuff is likely due to a variety of provider-specific reasons.

## Figures and Tables

**Table 1:** Percentage of cases with Endocuff by year.

	Gastroenterologists			Surgeons		
Year	SC	EAC	Total	SC	EAC	Total
2018	276,101 (99.5%)	1258 (0.45%)	277,359	70,202 (99.6%)	251 (0.36%)	70,453
2019	273,799 (99.5%)	1314 (0.48%)	275,113	69,340 (99.6%)	252 (0.36%)	69,592
2020	166,085 (99.0%)	1606 (0.96%)	167,691	46,515 (99.4%)	293 (0.63%)	46,808
2021	171,683 (99.0%)	1714 (0.99%)	173,397	47,826 (99.4%)	283 (0.59%)	48,109

EAC, Endocuff-assisted colonoscopy; SC, standard colonoscopy.

**Table 2:** Baseline characteristics for patients and providers by specialty type.

		*Gastroenterologists*			*Surgeons*		
		SC *N* = 887,669	EAC *N* = 5892	*p*-Value	SC *N* = 223,883	EAC *N* = 1079	*p*-Value
*Mean age ± SD, years*		60.4 ± 8.1	59.2 ± 7.8	<0.001	60.3 ± 8.1	59.7 ± 7.6	0.07
*Age, n (%)*							
	50–54	655,387 (73.8)	3803 (64.5)	<0.001	169,220 (72.4)	691 (64.0)	<0.001
	55–64	125,576 (14.1)	1195 (20.3)		35,595 (15.2)	228 (21.1)	
	65–69	53,754 (6.1)	493 (8.4)		14,925 (6.4)	91 (8.4)	
	70 and over	52,951 (6.0)	401 (6.8)		14,143 (6.0)	69 (6.4)	
*Gender, n (%)*							
	Female	464,908 (52.4)	3208 (54.4)	0.001	116,771 (49.9)	564 (52.3)	0.29
	Male	422,146 (47.6)	2684 (45.6)		117,087 (50.1)	515 (47.7)	
*Race, n (%)*							
	Black	122,129 (13.8)	1896 (32.2)	<0.001	23,702 (10.1)	330 (30.6)	<0.001
	Others	126,587 (14.3)	1035 (17.6)		18,773 (8.0)	68 (6.3)	
	White	638,952 (72.0)	2961 (50.3)		191,408 (81.8)	681 (63.1)	
*Endoscopist Experience Level, n (%)*				<0.0001			<0.0001
	≤500 cases	175,662 (19.8)	546 (9.3)		87,181 (37.3)	450 (41.7)	
	501–2000 cases	364,826 (41.1)	2630 (44.6)		118,826 (50.8)	628 (58.2)	
	2000 cases	347,180 (39.1)	2716 (46.1)		27,876 (11.9)	1 (0.1)	

EAC, Endocuff-assisted colonoscopy; SC, standard colonoscopy.

**Table 3:** Overall baseline characteristics for all patients, sensitivity cohort, and propensity score-matched cohort.

Variables		EAC Group (*N* = 6971)	SC Group			*p*-Value vs. EAC		
			All (*N* = 1,121,551)	Random Selection (*N* = 6971)	Propensity Score-Matched (*N* = 20,913)	SC All	SC Random Selection	SC Propensity Score-Matched
**Age, years**						<0.0001	<0.0001	<0.0001
	Mean ± SD	59.3 ± 7.8	60.4 ± 8.1	60.3 ± 8.1	61.0 ± 7.7			
**Age Group**						<0.0001	<0.0001	0.8006
	age 50–54	4494 (64.5)	824,607 (73.5)	5120 (73.4)	13,502 (64.6)			
	age 55–64	1423 (20.4)	161,171 (14.4)	974 (14.0)	4175 (20.0)			
	age 65–69	584 (8.4)	68,679 (6.1)	450 (6.5)	1781 (8.5)			
	age 70 and older	470 (6.7)	67,094 (6.0)	427 (6.1)	1455 (7.0)			
**Gender**						<0.0001	0.1269	0.8028
	Female	3772 (54.1)	581,679 (51.9)	3685 (52.9)	11,280 (53.9)			
	Male	3199 (45.9)	539,233 (48.1)	3284 (47.1)	9633 (46.1)			
**Race**						<0.0001	<0.0001	0.1703
	Black	2226 (31.9)	145,831 (13.0)	888 (12.7)	6441 (30.8)			
	Other	1103 (15.8)	145,360 (13.0)	926 (13.3)	3299 (15.8)			
	White	3642 (52.2)	830,360 (74.0)	5157 (74.0)	11,173 (53.4)			
**Hispanic**						<0.0001	<0.0001	<0.0001
	No	6246 (89.6)	883,541 (78.8)	5560 (79.8)	19,126 (91.5)			
	Unknown	311 (4.5)	159,351 (14.2)	928 (13.3)	851 (4.1)			
	Yes	414 (5.9)	78,659 (7.0)	483 (6.9)	936 (4.5)			
**Marital Status**						<0.0001	0.0027	0.6084
	Marry	4148 (59.5)	637,978 (56.9)	4026 (57.8)	12,307 (58.8)			
	Other	659 (9.5)	107,813 (9.6)	625 (9.0)	2064 (9.9)			
	Single	2152 (30.9)	371,751 (33.1)	2291 (32.9)	6513 (31.1)			
	Unknown	12 (0.2)	4009 (0.4)	29 (0.4)	29 (0.1)			
**Insurance Type**						<0.0001	<0.0001	0.8422
	Medicare	2001 (28.7)	360,008 (32.1)	2231 (32.0)	5936 (28.4)			
	Medicaid	407 (5.8)	94,894 (8.5)	612 (8.8)	1277 (6.1)			
	Commercial	4309 (61.8)	608,179 (54.2)	3785 (54.3)	12,930 (61.8)			
	Others	254 (3.6)	58,470 (5.2)	343 (4.9)	770 (3.7)			
**Bed Size**						<0.0001	<0.0001	<0.0001
	000–099	1203 (17.3)	177,875 (15.9)	1094 (15.7)	3742 (17.9)			
	100–199	1068 (15.3)	206,414 (18.4)	1294 (18.6)	3247 (15.5)			
	200–299	2230 (32.0)	149,994 (13.4)	910 (13.1)	6943 (33.2)			
	300–399	802 (11.5)	158,712 (14.2)	1011 (14.5)	1817 (8.7)			
	400–499	24 (0.3)	141,307 (12.6)	892 (12.8)	60 (0.3)			
	500+	1644 (23.6)	287,249 (25.6)	1770 (25.4)	5104 (24.4)			
**Region**						<0.0001	<0.0001	0.5703
	Midwest	437 (6.3)	386,803 (34.5)	2374 (34.1)	1396 (6.7)			
	Northeast	16 (0.2)	178,199 (15.9)	1096 (15.7)	58 (0.3)			
	South	5815 (83.4)	434,425 (38.7)	2749 (39.4)	17,329 (82.9)			
	West	703 (10.1)	122,124 (10.9)	752 (10.8)	2130 (10.2)			
**Teaching Hospital**						<0.0001	<0.0001	0.0332
	Yes	4617 (66.2)	618,762 (55.2)	3881 (55.7)	14,140 (67.6)			
	No	2354 (33.8)	502,789 (44.8)	3090 (44.3)	6773 (32.4)			
**Population Density**						<0.0001	0.0003	0.302
	Rural	1447 (20.8)	202,380 (18.0)	1278 (18.3)	4463 (21.3)			
	Urban	5524 (79.2)	919,171 (82.0)	5693 (81.7)	16,450 (78.7)			
**Endoscopist Experience**						<0.0001	<0.0001	0.1345
	≤500 cases	996 (14.3)	262,843 (23.4)	1549 (22.2)	3131 (15.0)			
	501–2000 cases	3258 (46.7)	483,652 (43.1)	3050 (43.8)	9886 (47.3)			
	>2000 cases	2717 (39.0)	375,056 (33.4)	2372 (34.0)	7896 (37.8)			
**Provider Specialty**						<0.0001	<0.0001	<0.0001
	Surgeons	1079 (15.5)	233,883 (20.9)	1432 (20.5)	4560 (21.8)			
	Gastroenterologists	5892 (84.5)	887,668 (79.1)	5539 (79.5)	16,353 (78.2)			

## Data Availability

The data presented in this study are available on request from the corresponding author.

## Supplementary Materials

The following supporting information can be downloaded at: https://www.mdpi.com/article/10.3390/jcm14176346/s1, Supplementary Table S1: Description of all ICD-10, HCPCS, and CPT codes utilized.
