# Supplementary material for: Use of Mechanical Enhanced Colonoscopy to Improve Polyp Detection During Colorectal Cancer Screening: A Real-World Healthcare Database Analysis
Source: J Clin Med. 2025 Sep 8;14(17):6346. doi: 10.3390/jcm14176346 (PMC12429699; doi:10.3390/jcm14176346)
Supplement: Supplementary file 1 [file jcm-14-06346-s001.zip › jcm-3790904-supplementary.pdf]

Supplementary Table 1: Description of all ICD-10, HCPCS, and CPT codes utilized

Code	Description
**Current Procedural Terminology (CPT)**	
45378	Colonoscopy, flexible, proximal to splenic flexure; diagnostic, with or without collection of specimen(s) by brushing or washing, with or without colon decompression (separate procedure)
45380	Colonoscopy, flexible; with biopsy, single or multiple
45381	Colonoscopy, flexible; with directed submucosal injection(s), any substance
45382	Colonoscopy, flexible; with control of bleeding, any method
45384	Colonoscopy, flexible; with removal of tumor(s), polyp(s), or other lesion(s) by hot biopsy forceps
45385	Colonoscopy, flexible; with removal of tumor(s), polyp(s), or other lesions by snare technique
45386	Colonoscopy, flexible; with transendoscopic balloon dilation
45388	Colonoscopy, flexible; with ablation of tumor(s), polyp(s), or other lesion(s) (includes pre- and post-dilation and guide wire passage, when performed)
45389	Colonoscopy, flexible; with endoscopic stent placement (includes pre- and post-dilation and guide wire passage, when performed)
45390	Colonoscopy, flexible; with endoscopic mucosal resection
45398	Colonoscopy, flexible; with band ligation(s) (eg, hemorrhoids)
**Healthcare Common Procedure Coding System (HCPCS)**	
G0105	Colorectal cancer screening; colonoscopy on individual at high risk
G0121	Colorectal cancer screening; colonoscopy on individual not meeting the criteria for high risk
**The International Classification of Diseases, Tenth Revision (ICD 10)**	
D12	Benign neoplasm of colon, rectum, anus and anal canal
D12.0	Benign neoplasm of cecum

D12.1	Benign neoplasm of appendix
D12.2	Benign neoplasm of ascending colon
D12.3	Benign neoplasm of transverse colon
D12.4	Benign neoplasm of descending colon
D12.5	Benign neoplasm of sigmoid colon
D12.6	Benign neoplasm of colon, unspecified
D12.7	Benign neoplasm of rectosigmoid junction
D12.8	Benign neoplasm of rectum
D12.9	Benign neoplasm of anus and anal canal
C18	Malignant neoplasm of colon
C18.0	Malignant neoplasm of cecum
C18.1	Malignant neoplasm of appendix
C18.2	Malignant neoplasm of ascending colon
C18.3	Malignant neoplasm of hepatic flexure
C18.4	Malignant neoplasm of transverse colon
C18.5	Malignant neoplasm of splenic flexure
C18.6	Malignant neoplasm of descending colon
C18.7	Malignant neoplasm of sigmoid colon
C18.8	Malignant neoplasm of overlapping sites of colon
C18.9	Malignant neoplasm of colon, unspecified
C19	Malignant neoplasm of rectosigmoid junction
C20	Malignant neoplasm of rectum
C21	Malignant neoplasm of anus and anal canal
C21.0	Malignant neoplasm of anus, unspecified
C21.1	Malignant neoplasm of anal canal
C21.2	Malignant neoplasm of cloacogenic zone
C21.8	Malignant neoplasm of overlapping sites of rectum, anus and anal canal
D01.0	Carcinoma in situ of colon
D01.1	Carcinoma in situ of rectosigmoid junction
D01.2	Carcinoma in situ of rectum
D01.3	Carcinoma in situ of anus and anal canal
K5.1418	Inflammatory polyps of colon with other complication
K51.4	Inflammatory polyps of colon
K51.40	Inflammatory polyps of colon without complications
K51.41	Inflammatory polyps of colon with complications
K51.411	Inflammatory polyps of colon with rectal bleeding
K51.412	Inflammatory polyps of colon with intestinal obstruction
K51.413	Inflammatory polyps of colon with fistula
K51.414	Inflammatory polyps of colon with abscess
K51.419	Inflammatory polyps of colon with unspecified complications
K63.5	Polyp of colon

Z12.11	Encounter for screening for malignant neoplasm of colon
Z80.0	Family history of malignant neoplasm of digestive organs
Z86.010	Personal history of colonic polyps
